# Successful conservative management of delayed perforation following endoscopic submucosal dissection of the esophagus: A case report

**DOI:** 10.1002/deo2.70115

**Published:** 2025-04-08

**Authors:** Sachiyo Onishi, Jun Takada, Kiichi Otani, Naoya Masuda, Hiroki Taniguchi, Kentaro Kojima, Masaya Kubota, Takashi Ibuka, Takuji Iwashita, Masahito Shimizu

**Affiliations:** ^1^ First Department of Internal Medicine Gifu University Hospital Gifu Japan

**Keywords:** conservative management, delayed perforation, drainage tube, enteral nutrition, esophageal endoscopic submucosal dissection (ESD)

## Abstract

Delayed perforation after esophageal endoscopic submucosal dissection is a rare complication that may result in severe outcomes. Here, we report a case of delayed perforation that was successfully managed with conservative treatment. A 72‐year‐old male with hypertensive renal failure and on maintenance hemodialysis underwent endoscopic submucosal dissection for a 2/3 circumferential superficial esophageal cancer in the middle thoracic esophagus, involving resection of 4/5 of the esophageal circumference. Locoregional steroid injections were administered after resection to prevent stenosis. No perforation occurred during the procedure; however, delayed perforation was identified on postoperative day 3. Endoscopy revealed necrosis and brittleness in a large area of the post‐endoscopic submucosal dissection ulcer. The patient developed fever and mediastinal emphysema, and endoscopic attempts to close the perforation were unsuccessful. Conservative management—including fasting, antibiotics, and subsequent drainage—was initiated. The patient's condition improved with drainage tube placement, enteral nutrition, and antibiotic administration. A follow‐up computed tomography scan on postoperative day 56 confirmed the resolution of mediastinal emphysema, and endoscopy revealed that the perforation healed with scarring. This case highlights that surgery may be avoided if appropriate treatment is initiated as early as possible, including drainage to prevent exposure to gastric and intestinal fluids, early initiation of enteral nutrition, rehabilitation to maintain strength, and blood transfusions as supportive care.

## INTRODUCTION

Endoscopic submucosal dissection (ESD), is a widely adopted technique for treating superficial esophageal cancer. It allows for en bloc resection, becoming a global standard.^1^, ^2^ However, serious complications like delayed perforation, although rare (0.1%), can be life‐threatening. Unlike intraoperative perforation, delayed perforation causes extensive necrosis throughout the entire digestive tract wall and requires emergency surgery. Incorrect diagnosis or delayed therapeutic intervention of this complication directly threatens the patient's life, and emergency surgeries for this condition carry significant risks.

We describe a case of successful conservative treatment for delayed perforation following esophageal ESD.

## CASE REPORT

**FIGURE 1:**
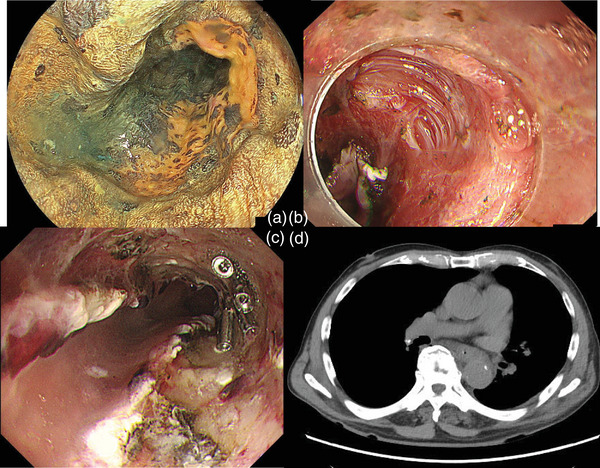
Endoscopic findings using Lugol's solution before treatment (a). Endoscopic findings showing muscle layer damage during endoscopic submucosal dissection (b). Endoscopic findings on the day after endoscopic submucosal dissection (c). Computed tomography scan on the day after treatment; no abnormalities other than the clip were observed (d).

The patient, a 72‐year‐old male, had a history of hypertensive renal failure on maintenance dialysis. During an esophagogastroduodenoscopy (EGD) at another hospital for screening, superficial esophageal cancer was detected, prompting a referral to our hospital for further evaluation and treatment. A detailed examination confirmed a 2/3 circumferential superficial esophageal cancer in the middle thoracic esophagus (Figure 1a), making the patient a candidate for ESD. The patient was subsequently admitted to the hospital for this procedure.

ESD was successfully performed, using a disposable high‐frequency knife: (IT‐nano KD‐612L; Olympus) and a standard electrosurgical generator (VIO 3; ERBE). The setting used included Dry Cut mode for incision and Swift Coag mode for dissection.　During the resection, muscularis propria damage was noted on the anal side of the resection area (Figure 1b), and endoscopic clipping was performed for closure. Due to the 4/5 circumferential resection, a locoregional steroid injection of Triamcinolone Acetonide (40 mg) was administered to prevent stenosis, with care taken to avoid injection near the exposed muscle layer and its surroundings.

**FIGURE 2:**
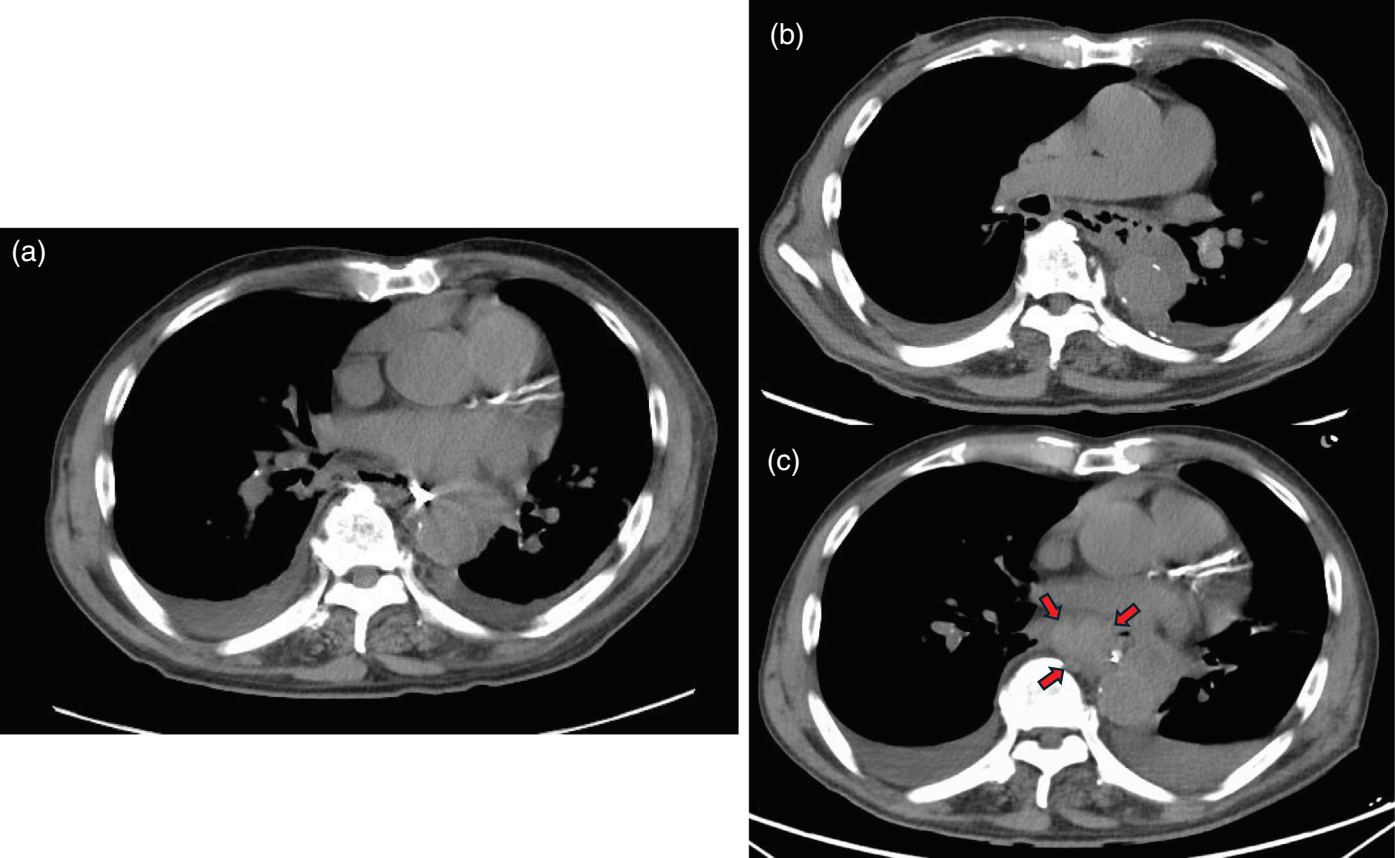
Findings at the time of diagnosis of delayed perforation. Computed tomography showed mediastinal emphysema and bilateral pleural effusion (a). Computed tomography scan just before placement of the water‐expelled drainage tube tube(b). Emergency computed tomography on postoperative day 16 showed the appearance of a hematoma in the mediastinum (red arrow) (c).

**FIGURE 3:**
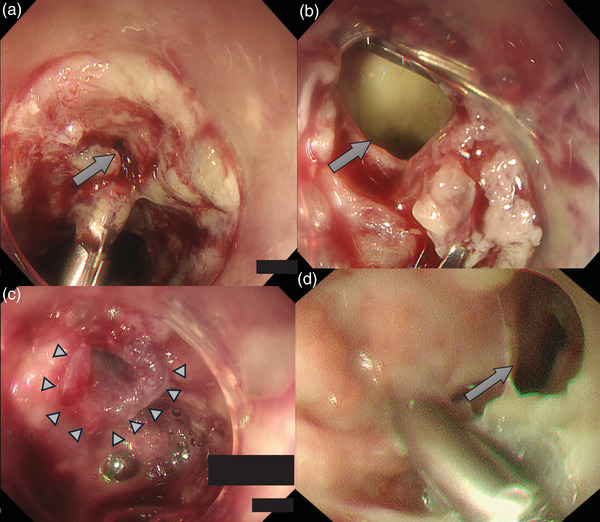
Endoscopic findings at the time of perforation discovery showed a small perforation near the clip (arrow) (a). Attempted clip closure resulted in an enlarged perforation (b). The polyglycolic acid sheet (arrowhead) successfully covered the perforation site (c), but it peeled off when the water‐expelled drainage tube tube was placed later and the post‐endoscopic submucosal dissection ulcer base was necrotic and prolapsed, revealing the mediastinum (arrow) (d).

On postoperative day 1 (POD1), the follow‐up EGD and computed tomography (CT) revealed no signs of perforation (Figure 1c,d and Table S1), and oral intake was resumed. However, on POD3, the patient developed a persistent fever exceeding 39°C. CT imaging revealed mediastinal emphysema (Figure 2a), and prompting an emergency EGD. Perforation was confirmed near the site of muscularis propria injury. Clip closure was attempted using a hemostasis clip (SureClip; Micro‐Tech); but the fragility of the ulcer base resulted in the perforation enlarging (Figure 3a,b). The use of a polyglycolic acid sheet to seal the perforation was considered but proved to be impractical in this case. The limited space made deployment of the sheet difficult, and the negative pressure within the mediastinum pulled the sheet inward, preventing effective coverage.

Considering the limitations of endoscopic treatment, in consultation with the surgical team, we decided to proceed with conservative treatment, including fasting and antibiotic therapy, taking into consideration the fact that the surgical procedure could not be determined because the pathological results of ESD were not yet available and the patient's overall condition was stable. However, there was a risk that the patient's condition would rapidly deteriorate, so we continued to monitor the patient's condition in consultation with the surgeon.

During conservative treatment, the patient's respiratory condition remained stable, and the fever gradually subsided. However, on POD7, a follow‐up CT scan revealed enlargement of the perforation (Figure 2b), and A water‐expelled drainage tube (wED) was inserted, with the side hole placed in the esophagus to drain gastric fluid. The tip of the catheter was positioned in the duodenal bulb to allow simultaneous enteral nutrition.

Endoscopic findings showed that the post‐ESD ulcer had become necrotic and sloughed over a wide area, primarily around the perforation site (Figure 3c). Nutritional management via the drainage tube and continued antibiotic treatment resulted in gradual improvement without worsening symptoms. Blood tests showed elevated inflammatory markers C‐reactive protein up to 15.7 mg/dL and white blood cell count up to 19,570/µL, but a gradual decrease in these values was observed thereafter. Rehabilitation was initiated early to maintain the patient's physical strength, alongside enteral nutrition, to support recovery.

On POD16, blood‐stained fluid was observed in the drainage tube, followed by a significant episode of hematemesis. Emergency CT imaging revealed the formation of a new hematoma in the mediastinum (Figure 2c). The patient developed worsening anemia necessitating a blood transfusion. As conservative treatment was considered insufficient, surgical intervention with subtotal esophagectomy was considered. However, coronary artery stenosis was detected during cardiac catheterization, indicating that emergency surgery carried a considerable risk. Given the patient's stable overall condition and resolution of fever, we decided to continue conservative management, supplemented by blood transfusions and ongoing enteral nutrition.

**FIGURE 4:**
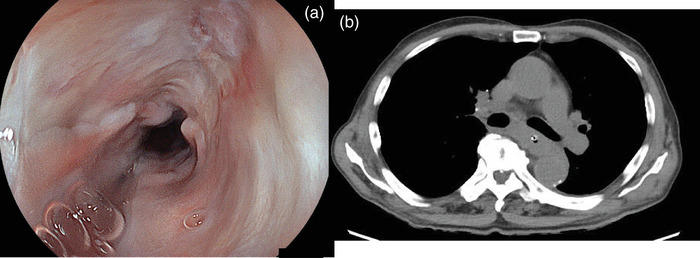
By postoperative day 60, esophagogastroduodenoscopy confirmed that the wound site had completely closed (a). The Computed tomography scan performed on postoperative day 57 showed that the pleural effusion and mediastinal emphysema had resolved (b).

Over time, bleeding from the drainage tube decreased, and a CT scan confirmed the resolution of the hematoma. The patient's general condition and blood test results improved. A CT scan on POD57 revealed no recurrence of mediastinal emphysema or hematoma (Figure 4b). On POD60, EGD confirmed that the fistula had healed with scarring, and the wED tube was removed (Figure 4a). The patient resumed oral intake without any recurrence of symptoms and was discharged on POD66 (Figure S1).

The pathological diagnosis of the resected specimen revealed well‐differentiated squamous cell carcinoma, 0‐IIc, pT1a‐LPM, INFa, Ly0, V0, HMX (unable to evaluate due to thermal denaturation), and VM0 (310 µm). The patient continued to be followed up in our outpatient clinic, with no evidence of recurrence or worsening of symptoms.

## DISCUSSION

Delayed perforation is a rare but serious complication of ESD, with a 0.1% incidence rate. Although intraoperative perforation occurs more frequently (1.8%),^2^ delayed perforation can be fatal without timely diagnosis and intervention. Given the negative pressure in the thoracic cavity, perforation leads to gastric fluid leaking into the mediastinum, causing chemical inflammation.^3^ This can be followed by bacterial infection, which may progress to mediastinal necrosis and carries a very high risk of severe septic shock. Mortality rates for esophageal perforation are approximately 20%,^4^ but prompt and appropriate early intervention can reduce the mortality rate to less than 50%, indicating that treatment timing is crucial.^4^ The cause of delayed perforation remains unknown, though excessive cauterization near the muscle layer may be a factor. Delayed perforation typically results in necrosis of the entire gastrointestinal wall, and emergency surgery is generally required.^5^ In this case, the patient is on maintenance hemodialysis, and having local steroid injections after ESD likely contributed to the delayed perforation. Hemodialysis patients often exhibit arteriosclerosis, ischemia, and hypoalbuminemia, increasing mucosal fragility and impaired tissue repair, heightening the risk of perforation.^6^, ^7^ The long‐lasting locoregional steroid may weaken the muscular layer, further increasing the risk of delayed perforation.^8^ Locoregional steroid injection is effective in preventing stenosis even if it is not immediately after ESD, and in the case of muscle layer injury, the risk of delayed perforation might have been a consideration to delay the timing of locoregional steroid injection.

Although emergency surgery is typically considered the only treatment for delayed perforation, early diagnosis, and prompt therapeutic intervention allowed conservative treatment in this case. Early enteral nutrition helps maintain immune function, promotes tissue repair, and regulates inflammatory responses, as the intestine is responsible for about 50% of the body's immune function.^9^ Maintaining appropriate nutritional status promotes tissue repair in the digestive tract and strengthens the overall immune response.^9^, ^10^ In this case, there were concerns regarding the long fasting period; however, early initiation of enteral nutrition, along with drainage, stabilized the patient's general condition. Nutritional management is considered a major factor in avoiding surgery.

The success factor was due to supportive care including enteral nutrition as early as possible, blood transfusions, rehabilitation, and timely medical intervention, all of which played a crucial role in the patient's recovery.

## CONFLICT OF INTEREST STATEMENT

None.

## Supporting information



FIGURE S1 Progress chart after hospitalization.

Supplement data table.docx
