# Supplementary figures and images for: Successful conservative management of delayed perforation following endoscopic submucosal dissection of the esophagus: A case report
Source: DEN Open. 2025 Apr 8;5(1):e70115. doi: 10.1002/deo2.70115 (PMC11977647; doi:10.1002/deo2.70115)

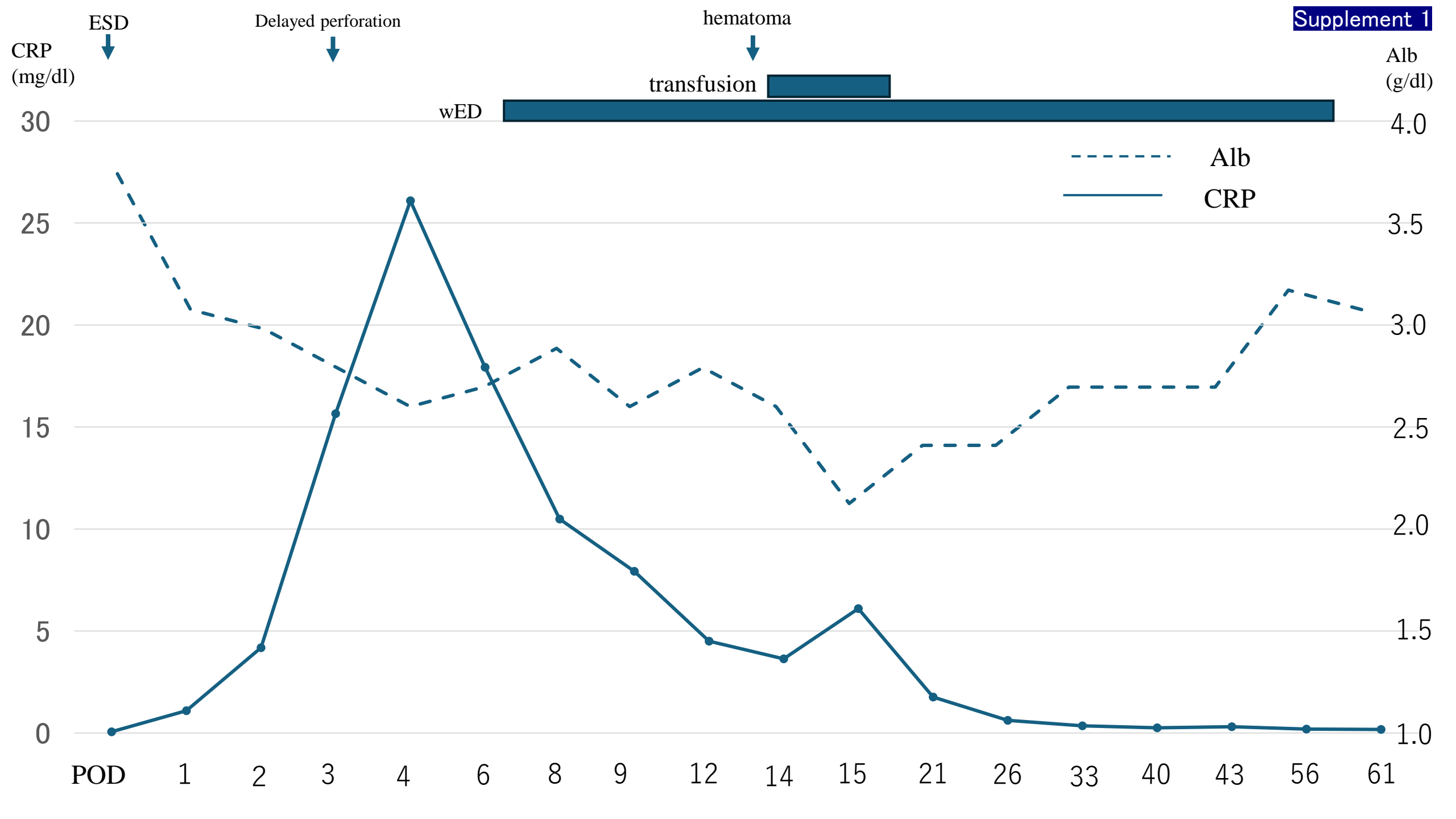

Supplement: Supplementary file 1 — FIGURE S1 Progress chart after hospitalization. [file DEO2-5-e70115-s001.pdf]
