# Supplementary material for: Successful conservative management of delayed perforation following endoscopic submucosal dissection of the esophagus: A case report
Source: DEN Open. 2025 Apr 8;5(1):e70115. doi: 10.1002/deo2.70115 (PMC11977647; doi:10.1002/deo2.70115)
Supplement: Supplementary file 2 — Supplement data table.docx [file DEO2-5-e70115-s002.docx]

| TP | 6.8 | g/dl |
| --- | --- | --- |
| Alb | 3.9 | g/dl |
| CK | 178 | U/L |
| AST | 15 | U/L |
| ALT | 16 | U/L |
| LD | 356 | U/L |
| ALP | 121 | U/L |
| γ-GT | 77 | U/L |
| ChE | 217 | U/L |
| Cre | 9.69 | mg/dL |
| UN | 54.8 | mg/dL |
| Na | 135 | mmol/L |
| K | 4.9 | mmol/L |
| Cl | 101 | mmol/L |
| T-Bil | 0.4 | mg/dL |
| CRP | 0.06 | mg/dL |
| WBC | 4.87× | 10^3^/μL |
| Hb | 12.8 | g/dL |
| Ht | 37.3 | % |
| MCV | 93 | fL |
| MCH | 31.9 | pg |
| MCHC | 34.3 | g/dL |
| Plt | 150× | 10^4^/μL |
| APTT | 28.2 | sec |
| PT-INR | 0.94 | % |

Supplement data 1: Data at the time of hospitalization

*TP: total protein, Alb: albumin, CK: Creatine Kinase, AST: aspartate aminotransferase, ALT: alanine aminotransferase, LD: lactate dehydrogenase, ALP: alkaline phosphatase, γGTP: γ glutamic pyruvic transaminase, ChE: cholinesterase, Cre: Creatinine, UN: urea nitrogen, Na: natrium, K: Kalium, Cl: chlorine, T-Bil: total bilirubin, CRP: C-reactive protein, WBC: white blood cell, Hb: hemoglobin, Ht: hematocrit, MCV: mean corpuscular volume, MCH: mean corpuscular hemoglobin, MCHC: mean corpuscular hemoglobin concentration, Plt: platelet, APTT: activated partial thromboplastin time, PT: prothrombin time
